# The Advantages of Inorganic Fertilization for the Mass Production of Copepods as Food for Fish Larvae in Aquaculture

**DOI:** 10.3390/life12030441

**Published:** 2022-03-17

**Authors:** Guo-Kai Hong, Kwee Siong Tew

**Affiliations:** 1 Graduate Institute of Marine Biology, National Dong Hwa University, Pingtung 944, Taiwan; 810663002@gms.ndhu.edu.tw; 2 National Museum of Marine Biology & Aquarium, Pingtung 944, Taiwan; 3 Institute of Marine Ecology and Conservation, National Sun Yat-sen University, Kaohsiung 80424, Taiwan; 4 International Graduate Program of Marine Science and Technology, National Sun Yat-sen University, Kaohsiung 80424, Taiwan

**Keywords:** copepod, fertilization method, aquaculture, *Pseudodiaptomus annandalei*, feed for larviculture, pathogens

## Abstract

Copepods are commonly used as live feed for cultured fish larvae, but the current mass production method using organic fertilizers cannot meet the market demand for copepods. We evaluated the feasibility of applying an inorganic fertilization method, which is currently in use in freshwater and marine larviculture, to the mass production of copepods. For 30 days, and with five replicates of each treatment, we made comparative daily measurements of various parameters of (1) copepod cultures fertilized with commercially available condensed fish solubles (organic fertilization) and (2) other cultures in which the concentration of inorganic phosphorus was maintained at 100 μg P L^−1^ and that of inorganic nitrogen at 700 μg N L^−1^ (inorganic fertilization). With inorganic fertilization, pH fluctuated over a smaller range and much less filamentous algae grew in the tanks. The mean production of copepod nauplii over the course of the study was similar between the two treatments, but the combined density of copepodites and adult copepods was significantly higher with inorganic fertilization. Compared to commercial zooplankton products, copepods cultured with inorganic fertilization were smaller, were mixed with fewer (almost none) non-copepod contaminants, were also pathogen-free, and could be produced at the cheapest cost per unit output. Based on these results, we conclude that the inorganic fertilization method can profitably be adopted by commercial copepod producers to meet the demand from fish farmers, especially for small-sized copepods.

## 1. Introduction

Aquatic organisms have become an increasingly important source of food protein for humans over the last few decades [1]. Per capita food fish consumption doubled from 9.0 kg (live weight equivalent) in 1961 to 20.5 kg in 2018 [1]. Because fish resources in the wild are quickly being exhausted, aquaculture is seen as the best way to meet growing demand. Total aquaculture production averaged a mere 14.9 million tons between 1986 and 1995 but reached 82.1 million tons in 2018 [1].

The provision of suitable prey items to fish larvae is a key factor for the success of aquaculture because undernutrition causes irreversible stunting [2]. Feeding them with a variety of naturally occurring live food items should enhance their survival rate in the early stages of development [3]. Artificial feeds have been developed, but for most fish larvae, live feed is still irreplaceable [4,5]. Various zooplankters, such as rotifers [6], copepods [7,8], and *Artemia* nauplii [9], attract the attention of fish larvae because of their movements [10,11].

Copepods are one of the most common live feeds for fish larvae [7]. They are widespread in the wild, and various indoor techniques for intensive culture have been developed [12,13]. Due to the high cost of indoor intensive culture [14,15], however, feed copepods are currently mainly sourced from the wild, from extensive culture outdoors, or as a by-product from aquaculture ponds [16,17,18]. Extensive culture of copepods outdoors often relies on traditional fertilization methods whereby different kinds of organic matter, such as animal manure [19], soybean meal [20], or alfalfa meal [21], are added to the culture pools to promote the growth of phytoplankton, which is then consumed by the copepods [19,22]. Such organic material varies in its nutrient content, sometimes resulting in the accumulation of excess nitrogenous waste when the pools are over-fertilized [23,24], or in low phytoplankton density when they are under-fertilized. Consequently, the organic fertilization method has not proven reliable in producing enough copepods to meet market demand.

An alternative method based on inorganic fertilization was first used in freshwater aquaculture in the 1990s [25,26,27]. It has since proved to be a reliable method for rearing freshwater percid larvae [28,29]. More recently, it has been applied to the larviculture of marine fish such as groupers and various coral-reef fish larvae, with some success [3,30,31]. In this method, liquid inorganic fertilizers are used to increase the nitrogen (N) and phosphorus (P) concentrations in the water. With regular monitoring to maintain constant nutrient concentrations and N:P ratios, it has proven possible to suppress the growth of filamentous blue-green algae while instead promoting the growth of small unicellular algae, which in turn enhanced zooplankton growth [25,28,30,31]. Analyses of the culture tank water and the stomach contents of newly hatched larval groupers grown under such an inorganic fertilization regime showed that copepods were the most abundant form of zooplankton and that they were actively consumed by fish larvae [30].

In the present study, we evaluated the feasibility of using this inorganic fertilization method for the mass production of copepods, by comparing it to the commonly used organic fertilization method. The objective was to develop a reliable method for providing live food to fish larvae and thus promote the large-scale rearing of other species of fish in aquaculture facilities in the future.

## 2. Materials and Methods

### 2.1. Experimental Design

The experiment was conducted at the National Museum of Marine Biology and Aquarium (NMMBA), Taiwan. Ten 1000 L round fiberglass tanks were placed outdoors and filled with unfiltered natural seawater from the adjacent coastal area. The time of sunrise and sun3set was about 0550 h and 1745 h, respectively. The light intensity at noon was about 2800 μmol m^−2^s^−1^. Five tanks (N = 5) were supplied daily with NH₄NO₃ and H₃PO₄ (Sigma-Aldrich, St. Louis, Missouri, USA) sufficient to maintain the following concentrations of inorganic nutrients: N, 700 μg L^−1^; P, 100 μg L^−1^. Commercially available condensed fish solubles (SINON Corporation, Taichung, Taiwan) were added daily as organic fertilizer to the other five tanks (N = 5) in quantities of 60, 30, 30, 30, and 30 mL per 1000 L over five successive days at the start of the experiment (a common procedure in local copepod production farms), but not thereafter. Adult calanoid copepods (*Pseudodiaptomus annandalei*, the dominant species in the local coastal area), were added to each tank at a density of 1 ind L^−1^ on Day 3. The experiment continued for 30 days.

### 2.2. Physicochemical Analyses

During the experiment, the temperature, salinity, dissolved oxygen (DO), and pH were monitored daily using a water quality instrument (YSI Professional Plus handheld multiparameter meter, YSI, Yellow Springs, OH, USA).

The nitrogen (NH_3_-N, NO_2_-N, and NO_3_-N) and phosphorus (PO_4_-P) concentrations were determined daily using HACH water analysis products (HACH, Loveland, CO, USA), including the NH_3_ kit (salicylate method 8155), NO_2_ kit (diazotization method 8507, with NO_3_ being reduced to NO_2_ beforehand [32]), and PO_4_ kit (ascorbic acid method 8048). Measurements were done using a spectrophotometer (Synergy H4 Hybrid Reader, BioTek Instruments, Winooski, VT, USA).

### 2.3. Biological Analyses

Water samples (200 mL) were collected daily from each tank and filtered through 0.45 μm membrane filters (Advantec, Tokyo, Japan) to obtain the phytoplankton. Each membrane was then extracted with 10 mL 90% acetone [33] and the chl a concentration was measured with a spectrophotometer (Hitachi U-5100, Hitachi, Tokyo, Japan).

Copepods as well as other zooplankton were collected daily by filtering 1 L of water from each tank through a 25 μm mesh plankton net. Zooplankters were categorized into two size groups (50–100 μm and > 100 μm), whereas copepods were classified into nauplii and adults (the latter including copepodites), and the respective densities were enumerated under a compound microscope. 

After counting the copepods, each sample was placed in an oven at 70 °C for three days to obtain the total copepod dry weight (adults + nauplii), which was measured using a 6-digit digital analytical microbalance (XP2U Ultra Micro Balance, Mettler-Toledo, Columbus, OH, USA). Commercially available frozen zooplankton bricks were purchased from four local fish farms (3 bricks per farm, total N = 12) to compare their zooplankton composition with our cultured samples, based on 500 individuals from each brick or each culture tank, and to compare their size distributions (total body length was measured), based on 50 randomly selected individuals from each sample, as well as the cost per unit dry weight (accumulation of total money spent on fertilizer/total dry weight).

On day 30, after we had collected all the zooplankton and drained the tanks, we also collected the filamentous yellow-green algae growing on the walls of the tanks, measured its wet weight, and after oven drying at 70 °C for three days, also measured its dry weight.

### 2.4. Statistical Analysis

The effects of different fertilization methods on physicochemical parameters, chl a concentration, total zooplankton density, copepod density, and total dry weight were analyzed with one-way repeated measures analysis of variance (RM-ANOVA) by treating sampling date as a repeated factor. The zooplankton sizes and cost per unit dry weight among different treatments and commercial products were analyzed with one-way ANOVA. Wet and dry weights of the filamentous algae produced as a by-product of inorganic and organic fertilization were compared using a *t*-test. All data were *ln*-transformed when necessary to meet the assumptions of normality and homogeneity of variance. Statistical computations were completed using SigmaPlot 12.5 (SPSS 1997); α = 0.05 was considered statistically significant.

## 3. Results

**Figure 1:**
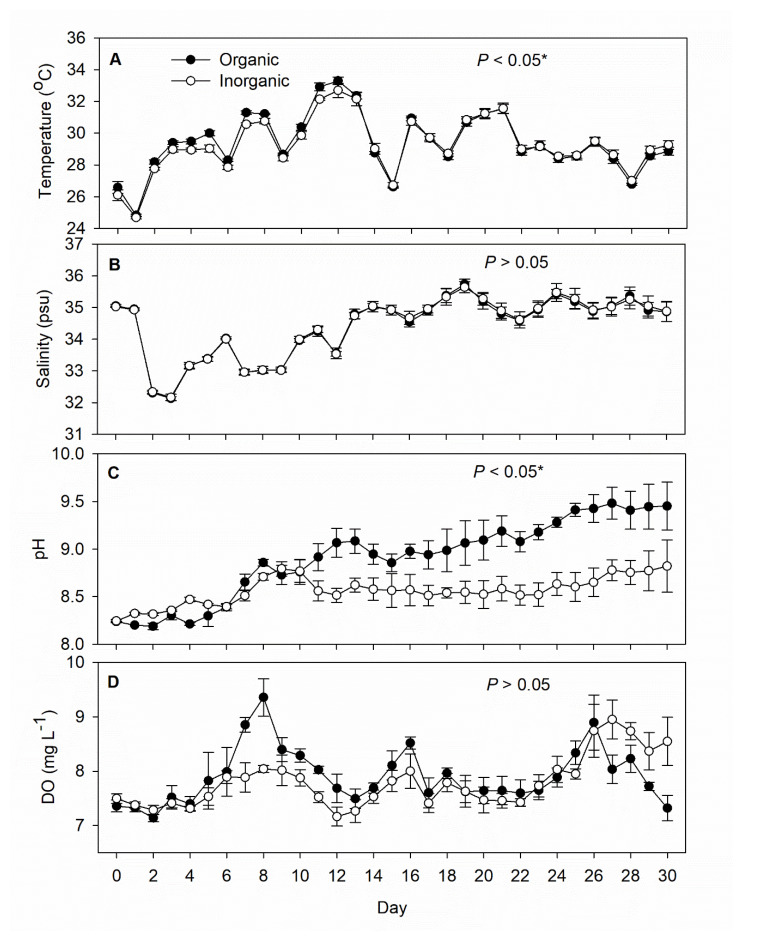
Daily records (mean ± SD) of (**A**) temperature, (**B**) salinity, (**C**) pH, and (**D**) dissolved oxygen in the inorganic (N = 5) and organic (N = 5) fertilization tanks during the 30-day experimental period. The *p*-value in each panel indicates the significance level (*, α = 0.05) of the treatment effect based on repeated measures ANOVA.

During the experiment, the water temperature in both treatments fluctuated essentially in tandem between 24 and 32 °C (*p* < 0.05), and the salinity similarly fluctuated in tandem between 32 and 35 psu (*p* > 0.05). Daily average pH increased from 8.2 at the start to 8.7–8.8 by Day 8 in both treatments, thereafter continuing to increase with organic fertilization to 9.0 after 10 days and almost 9.5 after 20 days, while remaining rather steady at 8.5–8.7 under inorganic fertilization until a slight rise at the end. Overall, the mean pH under inorganic fertilization was 8.56 ± 0.15, significantly lower than the mean of 8.91 ± 0.42 observed under organic fertilization (*p* < 0.05). DO measurements were similar overall between the two treatments (*p* > 0.05) (Figure 1).

**Figure 2:**
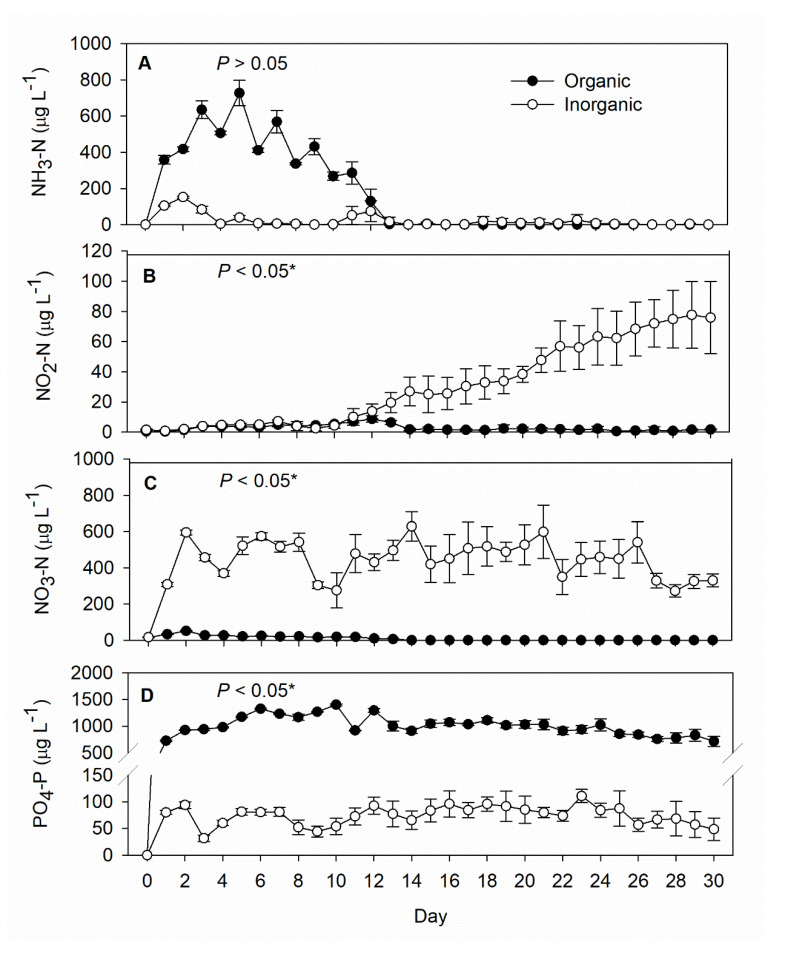
Daily records (mean ± SD) of (**A**) ammonium-nitrogen (NH_3_-N), (**B**) nitrite-nitrogen (NO_2_-N), (**C**) nitrate-nitrogen (NO_3_-N), and (**D**) phosphate (PO_4_-P) concentrations in the inorganic (N = 5) and organic (N = 5) fertilization tanks during the 30-day experimental period. The *p*-value in each panel indicates the significance level (*, α = 0.05) of the treatment effect based on repeated measures ANOVA.

The initial nutrient concentration was very low in both treatments (Figure 2). After fertilization had begun, the mean concentration of NH_3_-N in the inorganic treatment tanks rose overnight to 105 ± 4 µg L^−1^, then to 152 ± 6 µg L^−1^ on Day 2, but after just one more day at 84 ± 19 µg L^−1^, dropped to less than 50 µg L^−1^ (usually not exceeding 20 µg L^−1^) for the remainder of the experiment. In the organic treatment tanks, on the other hand, NH_3_-N rose to 358 ± 24 µg L^−1^ overnight and remained higher than 100 µg L^−1^ through Day 12, after which it never again exceeded 10 µg L^−1^ (Figure 2A).

The NO_2_-N concentration was initially low in the organic treatment tanks but gradually increased to more than 70 µg L^−1^ (Figure 2B) by the end of the study. The NO_3_-N concentration was maintained at a mean of 450 ± 102 µg L^−1^ in the inorganic treatment tanks during the course of the experiment, significantly higher than with the organic treatment (10 ± 13 µg L^−1^) (Figure 2C). The mean PO_4_-P concentration throughout the experiment was significantly (about 15 times) lower with the inorganic treatment (74 ± 18 μg L^−1)^ than the organic treatment (1009 ± 179 µg L^−1)^ (Figure 2D).

**Figure 3:**
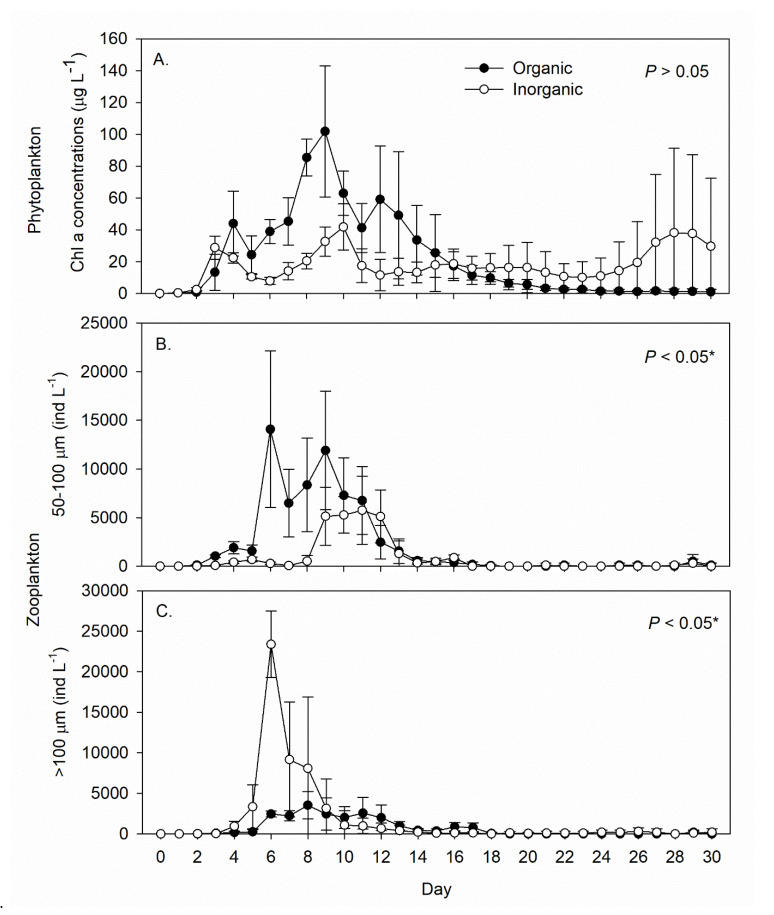
Daily records (mean ± SD) of (**A**) phytoplankton chlorophyll a concentration and (**B**,**C**) zooplankton abundance in the inorganic (N = 5) and organic (N = 5) fertilization tanks during the 30-day experimental period. The *p*-value in each panel indicates the significance level (*, α = 0.05) of the treatment effect based on repeated measures ANOVA.

Phytoplankton started to bloom in the tanks three days after the first fertilization in both treatments (Figure 3A). The chl a concentration peaked in the organic treatment tanks on Day 9 (102 ± 41 µg L^−1^), then declined gradually to less than 10 µg L^−1^ after Day 17. In the inorganic treatment tanks, the chl a concentration attained 29 ± 7 µg L^−1^ on Day 3 and remained higher than 10 µg L^−1^ for the remainder of the experiment (Figure 3A). The mean wet and dry weights of the filamentous algae (*Tribonema* sp.) collected from all the tanks on Day 30 were 2464 ± 164 and 184 ± 12 g tank^−1^, respectively, for the organic fertilization treatment, but only 1.4 ± 0.2 and 0.10 ± 0.02 g tank^−1^, respectively, for the inorganic fertilization treatment.

Zooplankton abundance (excluding copepods) in the 50–100 µm size range was significantly higher with organic fertilization, especially between Days 6 and 11 (Figure 3B). This size class consisted primarily (>90%) of ciliates, i.e., *Euplotes* spp. As for the > 100 µm size range, *tintinnid* ciliates and *Strombidium* spp. thrived under inorganic fertilization during Days 5–9 (Figure 3C).

**Figure 4:**
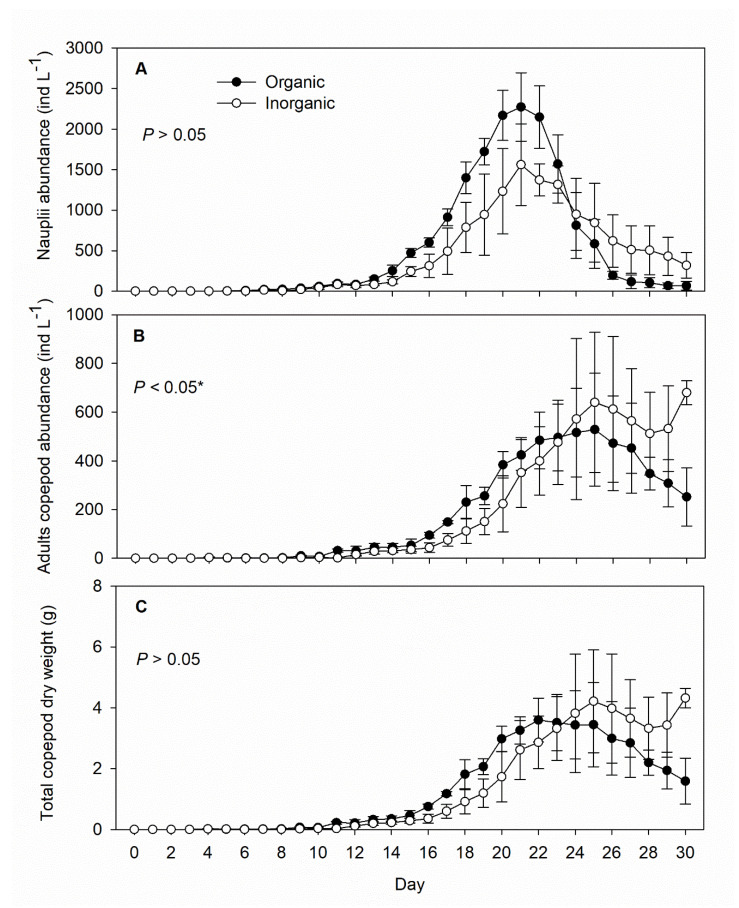
Daily records (mean ± SD) of abundance (density) of (**A**) copepod nauplii and (**B**) copepodites/adults and (**C**) copepod dry weight in the inorganic (N = 5) and organic (N = 5) fertilization tanks during the 30-day experimental period. The *p*-value on each panel indicates the significance level (*, α = 0.05) of the treatment effect based on repeated measures ANOVA.

In both the organic and inorganic treatment tanks, copepod nauplii started to appear on Day 6, peaked on Day 21, and declined thereafter (Figure 4A). Under both treatments, the combined copepodites and adults peaked 5 days after the nauplii, and while the combined density of copepodites and adults in the organic treatment tanks then declined, their density remained significantly higher in the inorganic treatment tanks (Figure 4B). Copepod dry weight, including nauplii, showed a similar pattern to copepodite/adult density (Figure 4C).

**Figure 5:**
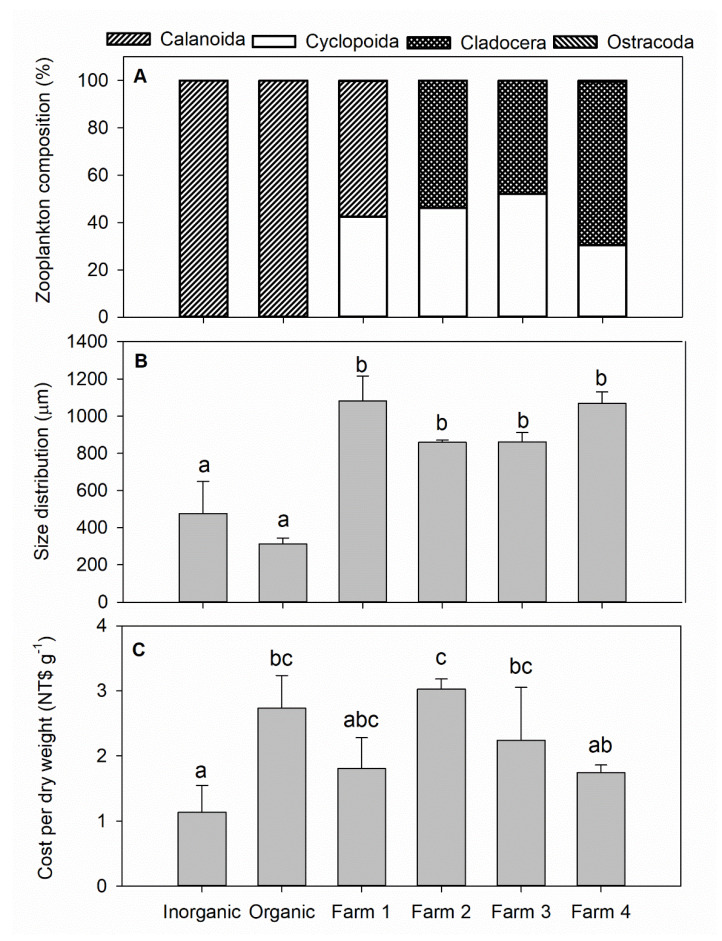
Comparison of (**A**) composition (N = 500 each), (**B**) size distribution (N = 50 each), and (**C**) cost per dry weight among zooplankton samples from the experimental inorganic (N = 5) and organic (N = 5) fertilization tanks and four commercial products (N = 3 each). Bars in a panel that share the same lower-case letters indicate no statistical difference among sources (*p* > 0.05).

The zooplankton in the organic and inorganic treatment tanks was composed almost entirely of calanoid copepods, whereas the commercial products were mixtures of cyclopoid copepods, calanoids, cladocerans, and ostracods (Figure 5A). The zooplankton size distributions in both the inorganic and organic treatment tanks were similar to each other on Day 21, but significantly smaller than in all four of the commercial products (Figure 5B), which were similar among themselves. The cost per dry weight of zooplankton from the inorganic treatment tanks was the lowest among all samples, being significantly cheaper to produce than zooplankton from the organic treatment tanks (the second most expensive) and less than the cost of two of the commercial products (Figure 5C).

## 4. Discussion

Providing larval fish with a suitable selection and quantity of live feed is essential to the survival of marine and freshwater fish in aquaculture [34,35]. Various zooplankters, such as rotifers [6], copepods [7,8], and *Artemia* nauplii [9], are commonly used in larviculture. Some other larger zooplankton such as mysids, amphipods, and ostracods are sometimes very abundant in the natural environment and are thus eaten by fish larvae. Artificial feeds have been developed but are less successful than live food for culturing most species of fish larvae [4,5].

Among the different types of live feed currently available for larval fish aquaculture, copepods have been shown to be excellent for the larvae of many fish species [35,36,37,38]. Nonetheless, no standard procedure has yet been adopted in the aquaculture industry for the mass production of copepods. Commercially available copepods, at least in Taiwan, are mostly produced by extensive culture outdoors or as a by-product in aquaculture ponds [16,17,18]. Our results show that the density, as well as the composition and size distribution of such commercial copepod products vary from pond to pond and from time to time, as well as among different farms.

In this study, we used inorganic fertilizer to grow copepods and we compared the results to those obtained using condensed fish solubles, a more commonly applied organic fertilizer. During the 30 days of the study, fluctuations in dissolved oxygen concentration were similar between the inorganic and organic treatments, whereas pH was significantly higher in the latter. The highest recorded pH value of about 9.5, which persisted in the organic treatment tanks during the last week of the study, could represent a danger to copepods, some species of which produce no eggs at pH 9.5 [39]. In some other copepod species, the mortality of nauplii gradually increases with an increase in pH from 9.0 to 9.5 [39]. A pH of 9.5 also significantly reduced the protozooplankton biomass and diversity, and thus the potential food supply for copepod larvae in a natural marine planktonic community [40]. Failure of many nauplii to grow into copepodites and adult copepods in the organic treatment tanks might be attributed to high pH.

Nutrient concentrations, especially of NH_3_-N and PO_4_-P, increased dramatically after fertilization with concentrated fish solubles. Previous work has shown that the hatching success of eggs of the calanoid copepod *Acartia clausi* decreased by 50% after a 9-day exposure to 98 μg L^−1^ NH_3_-N [41], while the LC_50_ of NH_3_-N was 1035 μg L^−1^ (48 h) for larvae of *A. tonsa*, and 800 μg L^−1^ (48 h) and 634 μg L^−1^ (72 h) for adults of that species [42]. Excess phosphate has been reported to enhance the growth of the benthic filamentous algae *Tribonema* spp. [43,44], and a very low N:P ratio reportedly favors the growth of filamentous algae in freshwater aquaculture ponds [25,26,28]. In the present study, in which the PO_4_-P concentration remained 10 times higher under organic fertilization than under inorganic fertilization through the entire course of the experiment and N was very low after Day 12, the tanks into which organic fertilizer was applied (but not the others) indeed became overgrown with such algae. Overgrowth of filamentous algae may have also suppressed the growth of phytoplankton in those tanks, thereby possibly contributing to the relative failure of development of nauplii to adult copepods under organic fertilization by reducing the copepods’ supply of food. The precisely adjusted nutrient concentrations and N:P ratio afforded by the inorganic fertilization method probably inhibited the filamentous algae and encouraged the growth of unicellular algae in those tanks [3,30,31], thereby providing better conditions for the growth of copepod nauplii.

Phytoplankton serves as an intermediary between nutrients and copepods in terms of energy transfer. A continuous supply of the right amount and ratio of nutrients will sustain a stable density of phytoplankton, which then supports the growth of copepods. We thought it odd, therefore, that with inorganic fertilization, the densities in culture of both the phytoplankton and the copepodite/adult copepods rose a second time near the end of the experiment while the density of nauplii continued to decline. At that time, large numbers of *Nitzschia* sp. growing in clusters were present in the inorganic treatment tanks. The exudates of this diatom consist of high-molecular-weight carbohydrates [45] that can inhibit grazing by predators. Furthermore, excessive consumption of diatoms has a negative effect on the reproduction of zooplankton [46,47]. In the present case, the nauplii that were already present continued to grow into adults, but recruitment of new offspring was impaired [46]. Occasional inoculation of other types of phytoplankton during the culturing period might forestall or prevent such inhibition of copepod recruitment. An experiment to test this idea should be carried out in the future.

After the addition of fertilizer, many non-copepod zooplankters such as ciliates grew in both sets of experimental tanks, with *Strombidium* spp. (~100–120 μm) in the inorganic fertilizer series and *Euplotes* spp. (~50–100 μm) in the organic fertilizer series. These contaminant zooplankton must be monitored because they may compete with copepods for phytoplankton [48,49]. Since ciliate community composition is correlated with aquatic habitat conditions, and particular species thrive only in an environment that is favorable for them [50], similar ciliates will likely dominate the culture-tank population whenever a similar fertilization method is used for growing the copepods. *Strombidium* spp. have the potential to become biological control agents in aquaculture because they actively remove bacterial pathogens [51]. In fact, when we checked for disease organisms and pathogens in the final products from our experimental treatments and compared them to commercial zooplankton products, we found only in the latter a microsporidian parasite, *Enterocytozoon hepatopenaei* (EHP) [52], and the bacterium *Vibrio parahaemolyticus* that causes Acute Hepatopancreatic Necrosis Disease (AHPND) in cultured shrimp [53] in the commercial products but not in our samples (data not shown). The fact that our experimental tanks were pathogen-free, in contrast to the commercial products, suggests that there may be a benefit to the presence of at least certain ciliates, especially *Strombidium* spp., that develop along with copepods as a result of employing the inorganic fertilization method.

In summary, after 15 days of cultivation, the composition of the zooplankton cultures resulting from our two different fertilization methods were uniformly almost 100% calanoid nauplii and small calanoid copepodites and adults, whereas the commercial products were mixtures of larger crustaceans such as adult cyclopoids, calanoids, cladocerans, and ostracods. The production cost of using inorganic fertilization was the lowest among all options, with little development of filamentous algae in the tanks and no bacterial pathogens. Inorganic fertilization may, therefore, be more than an effective method for fish larviculture, but also potentially very useful for the mass production of live feed such as copepods for fish larvae.

## Data Availability

Not applicable.
